# T182. SHARED AND DISTINCT ALTERATIONS IN THE WHITE MATTER TRACTS OF REMITTED AND NON-REMITTED PATIENTS WITH SCHIZOPHRENIA

**DOI:** 10.1093/schbul/sby016.458

**Published:** 2018-04-01

**Authors:** Tzung-Jeng Hwang, Jing-Ying Huang, Chih-Min Liu, Yu-Jen Chen, Yung-Chin Hsu, Hai-Gwo Hwu, Yi-Tin Lin, Ming-Hsien Hsieh, Chen-Chung Liu, Yi-Ling Chien, Wen-Yih Isaac Tseng

**Affiliations:** 1 National Taiwan University Hospital; 2 National Taiwan University

## Abstract

**Background:**

Antipsychotic drugs are the standard treatment for schizophrenia; however, the treatment outcomes vary. Different treatment outcomes may be attributed to the genetic and molecular heterogeneity of patients, which may be represented in the white matter structures of the brain. In the present study, we assessed the association between white matter tract integrity and treatment outcomes in patients with schizophrenia.

**Methods:**

We evaluated 96 patients with schizophrenia (remitted, 53; non-remitted, 43) and 50 healthy controls through diffusion spectrum imaging with a 3 Tesla magnetic resonance imaging scanner. Patients were categorized into the remission and non-remission groups according to the criteria proposed by The Remission in Schizophrenia Working Group (RSWG) on the basis of PANSS scores. White matter tract integrity was assessed through an automatic tract-specific analysis method to determine the mean generalized fractional anisotropy (GFA) values of the 76 white matter tract bundles in each participant.

**Results:**

Analysis of covariance revealed that 7 tracts, namely the bilateral fornices, the bilateral uncinate fasciculi, and the callosal fibers (CFs) of the bilateral temporal poles, bilateral hippocampi, and bilateral amygdalae, had significantly different GFA values among the 3 groups. Posthoc between-groups analysis showed that the non-remission group had lower GFA values in all 7 tracts than the control group; the remission group had lower GFA values than the control group only in 4 tracts, namely the bilateral fornices and the CFs of the bilateral temporal poles, and bilateral hippocampi. Compared with the remission group, the non-remission group had lower GFA values in all 7 tracts.

**Discussion:**

All 7 tracts that were altered in the non-remission group are a part of the limbic system, which supports various functions, including emotions, memory, and learning. Our results suggest that patients who had poor outcomes to antipsychotic treatments might have more severe disruptions in the limbic system. The 7 altered tracts in the non-remission group are compatible with those reported in previous studies on white matter or gray matter alterations. In a cross-sectional tractography-based study on 3 pairs of association fibers (i.e., the cingulum, superior longitudinal fasciculus, and uncinate fasciculus), Luck et al reported that compared with patients with good outcomes, patients with poor outcomes had reduced FA in the uncinate fasciculus and superior longitudinal fasciculus. Marques et al performed a longitudinal study using tract-based spatial statistics and reported that non-responders had more tracts with a significantly lower FA than did the responders, particularly in the uncinate fasciculus and corpus callosum. In addition to the uncinate fasciculus, we also observed reduced fiber integrity in the bilateral fornices and the CFs of the bilateral temporal poles, bilateral hippocampi, and bilateral amygdalae; these tracts connect the gray matter in the limbic system. Jääskeläinen et al revealed that a reduction in gray matter volume in the frontal and limbic areas is associated with overall poor outcomes. In addition, Van Haren et al reported significantly reduced gray matter volumes in the frontal and temporal cortices of the individuals with poor outcomes. Because the gray matter regions are anatomically connected by the fiber tracts, gray matter reduction in the limbic system might affect the interconnecting fiber tracts; this finding accords with the findings of the present study.

In conclusion, differences in the severity of white matter tract alterations in the remission and non-remission groups might indicate biologically distinct subgroups in schizophrenia.

